# Development and validation of a predictive model for stroke associated pneumonia in patients after thrombectomy for acute ischemic stroke

**DOI:** 10.3389/fmed.2024.1370986

**Published:** 2024-03-05

**Authors:** Jingying Wang, Chao Yang, Ruihai Zhang, Wei Hu, Peng Yang, Yiqing Jiang, Weijun Hong, Renfei Shan, Yongpo Jiang

**Affiliations:** ^1^Department of Emergency Medicine, Taizhou Hospital of Zhejiang Province Affiliated to Wenzhou Medical University, Taizhou, China; ^2^Department of Neurosurgery, Taizhou Hospital of Zhejiang Province Affiliated to Wenzhou Medical University, Taizhou, China; ^3^Department of Neurology, Taizhou Hospital of Zhejiang Province Affiliated to Wenzhou Medical University, Taizhou, China; ^4^Department of Critical Care Medicine, Taizhou Hospital of Zhejiang Province Affiliated to Wenzhou Medical University, Taizhou, China

**Keywords:** acute ischemic stroke, thrombectomy, stroke-associated pneumonia, risk factors, prediction model

## Abstract

**Objective:**

This study aims to identify the risk factors associated with stroke-associated pneumonia (SAP) in patients who have undergone thrombectomy for acute ischemic stroke and to develop a nomogram chart model for predicting the occurrence of pneumonia.

**Methods:**

Consecutive patients who underwent thrombectomy for acute ischemic stroke were enrolled from three hospitals at Taizhou Enze Medical Center. They were randomly divided into a training group and a validation group in a 7:3 ratio. The training group data was used to screen for effective predictive factors using LASSO regression. Multiple logistic regression was then conducted to determine the predictive factors and construct a nomogram chart. The model was evaluated using the validation group, analyzing its discrimination, calibration, and clinical decision curve. Finally, the newly constructed model was compared with the AIS-APS, A2DS2, ISAN, and PANTHERIS scores for acute ischemic stroke-associated pneumonia.

**Results:**

Out of 913 patients who underwent thrombectomy, 762 were included for analysis, consisting of 473 males and 289 females. The incidence rate of SAP was 45.8%. The new predictive model was constructed based on three main influencing factors: NIHSS ≥16, postoperative LMR, and difficulty swallowing. The model demonstrated good discrimination and calibration. When applying the nomogram chart to threshold probabilities between 7 and 90%, net returns were increased. Furthermore, the AUC was higher compared to other scoring systems.

**Conclusion:**

The constructed nomogram chart in this study outperformed the AIS-APS, A2DS2 score, ISAN score, and PANTHERIS score in predicting the risk of stroke-associated pneumonia in patients with acute ischemic stroke after thrombectomy. It can be utilized for clinical risk prediction of stroke-associated pneumonia in patients after thrombectomy for acute ischemic stroke.

## Introduction

Stroke-associated pneumonia (SAP) is one of the most common complications in stroke patients. It is a pulmonary infection that occurs during the acute and sequelae phases of stroke (1). Stroke has a high incidence and mortality rate, with survey data showing approximately 13.7 million new stroke cases worldwide (2). Among these cases, stroke-associated pneumonia accounts for 7–22%, and the number of deaths attributable to stroke reaches up to 2 million every year (2). Studies have shown that pneumonia triples the risk of 30-day mortality in stroke patients (3). At the present, various studies have found that the pathophysiology of stroke-associated pneumonia is mainly related to the inflammatory mechanism and aspiration (4, 5). The systemic immunosuppression that occurs during the acute phase of stroke, known as post-stroke immunosuppressive syndrome, increases susceptibility to bacterial infections and is one of the major causes of infection in patients (6–8). The occurrence of stroke-associated pneumonia can cause a systemic inflammatory response, exacerbate neurological damage in stroke patients, prolong their hospitalization time, increase hospitalization costs and economic pressure, and lead to a poor prognosis, thereby increasing the probability of patient death (3, 9, 10). Early identification and treatment of stroke-associated pneumonia is of great significance for the prognosis of stroke patients.

Previous studies comparing the prognosis of thrombectomy and thrombolysis in patients with acute ischemic stroke have shown that thrombectomy alone is not inferior in improving functional outcomes compared to patients who underwent remove thrombus after thrombolysis within 4.5 h after symptom onset (11). Thrombectomy is the main treatment method for patients with large vessel stroke nowadays (12). With the increasing number of stroke patients undergoing thrombectomy, early warning of stroke-associated pneumonia in thrombectomy patients is becoming more important.

Presently, various scoring scales and inflammatory indicators are used in clinical practice to predict the occurrence of stroke-associated pneumonia. The AIS-AP score was proposed in 2013 through a study of the Chinese Stroke Login Database. It includes age, history, mRS (modified Rankin Scale) score, NIHSS (National Institutes of Health Stroke Scale) score, GCS (Glasgow Coma Scale) score, speech impairment, stroke classification, and blood sugar, but scoring using this scale is relatively cumbersome (13). The A2DS2 score was proposed based on a study of 15,335 registered stroke cases in Berlin, Germany. The score includes five items: gender, age, atrial fibrillation, dysphagia, and admission NIHSS score (14). The ISAN score was collected from 23,199 stroke patients in the UK and was scored based on four factors: age, gender, admission NIHSS, and ability to take care of oneself before the stroke (15). The PANTHERIS score was studied on 335 stroke patients in the intensive care unit of the hospital. The score includes four items: patient age, GCS upon admission, 24-h systolic and systolic blood pressure upon admission, and WBC (white blood cell) count (16). However, due to the differences in the samples studied by these scores and the fact that the sample study subjects are stroke patients, not targeted at stroke thrombus removal patients, there is no consensus on which score is more accurate in predicting SAP. Therefore, the main purpose of this study is to develop and validate a prediction model for SAP in patients after stroke thrombectomy. This model will serve as a basis for early clinical treatment, enabling healthcare providers to identify patients at risk and initiate timely interventions (17).

## Methods

### Participants

**Figure 1:**
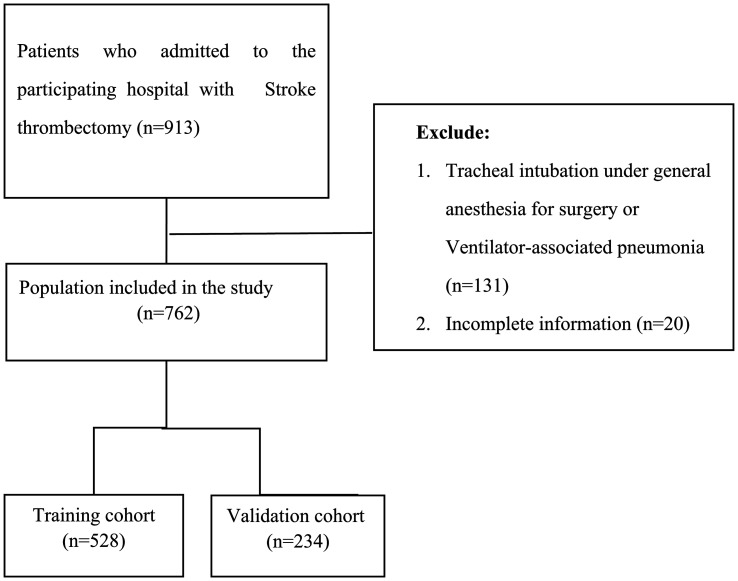
Study flow diagram.

This study is a retrospective study involving 913 consecutive patients with acute ischemic stroke who underwent thrombectomy at Taizhou Enze Medical Center from January 2021 to August 2023. Out of these, 762 patients were selected and randomly divided into a training set of 528 and a validation set of 234 at a 7:3 ratio (see Figure 1). The research proposal has been reviewed by the Ethics Committee of Taizhou Enze Medical Center (Group) (Ethics Number: K20201104). **Inclusion criteria**: (1) Patients who meet the diagnostic criteria for acute ischemic stroke in the 2018 Early Management Guidelines for Acute Ischemic Stroke (18), with an onset time of less than 24 h; and (2) patients who undergo mechanical thrombectomy. **Exclusion criteria**: (1) Cerebral hemorrhage and intracranial mass; (2) Transient ischemic attack; (3) Existence of immune system and blood system diseases; (4) Serious infection present before admission; (5) Patients automatically discharged or dying within 24 h of admission; (6) Liver and kidney failure; (7) Trauma; (8) Pregnant women or under the age of 18; (9) Tracheal intubation under general anesthesia for surgery or Ventilator-associated pneumonia; and (10) Patients with incomplete data collection. We applied for an informed consent waiver while keeping patient information confidential.

Clinical data collection and grouping: Patient information will be collected through the hospital’s medical record system, including age, gender, past medical history (diabetes, hypertension, cerebral infarction, atrial fibrillation, heart failure, COPD (Chronic obstructive pulmonary disease), smoking history), GCS score, NIHSS score on admission, systolic pressure on admission, thrombolysis, Blockage of blood vessels, tracheal intubation, dysphagia and blood test indicators [WBC, Lym (lymphocyte count), Neu (neutrophil count), Mono (mononuclear leucocyte), PLT (platelet), Hb (hemoglobin), FBG (fasting blood glucose), PT (prothronmbin time), INR (international normalized ratio), APTT (activated partial thromboplastin time), FIB (fibrinogen), TT (thrombin time), D-dimer, CL (chloride), NA (sodium), ALB (albumin)]. Additionally, the patient’s LMR (lymphocyte/monocyte ratio), NLR (neutrophil/lymphocyte ratio), SII (platelet * neutrophil/lymphocyte count), PLR (platelet/lymphocyte ratio), SIS (inflammation score, a comprehensive score of ALB and LMR) (19), as well as AIS-APS score, A2DS2 score, ISAN score, and PANTHERIS score will be calculated based on the collected information. The laboratory indicators obtained by the patient within 24 h before surgery are used as preoperative indicators. The laboratory results 24 h after surgery are used as postoperative indicators.

### Definition and indicators of pneumonia

The improved diagnostic criteria for stroke-associated pneumonia are based on the Centers for Disease Control and Prevention (CDC) guidelines (20). (1) Stroke-associated pneumonia refers to lower respiratory tract infections that occur within the first week (7 days) after a stroke in terms of onset time. (2) Diagnosis must comply with the modified SAP standards proposed by the US CDC, which include two levels: suspected SAP (compliance with CDC standards without typical chest X-ray changes even in repeated or continuous examinations) and confirmed SAP (compliance with CDC standards, including typical chest X-ray changes). (3) The diagnostic value of white blood cell count or C-reactive protein (CRP) for stroke-associated pneumonia is limited. (4) There is not enough evidence to suggest that other biomarkers, such as procalcitonin (PCT), are meaningful for the diagnosis of stroke-associated pneumonia. According to the standards of the CDC in the United States, patients are classified into SAP group and non-SAP group based on the occurrence of stroke-associated pneumonia.

### Study procedure

The data collected and organized were analyzed using LASSO regression to determine the factors that influence stroke-associated pneumonia in patients undergoing acute ischemic stroke thrombectomy. Subsequently, stepwise regression analysis was performed to obtain a multivariate logistic regression model. The statistically significant factors (*p* < 0.05) identified in the model included NIHSS score ≥ 16, postoperative LMR, and dysphagia. These factors were included in the column chart to establish the model. After constructing the model, internal validation was conducted using the training set, and external validation was carried out using the validation set. The discriminability and calibration of the column chart were evaluated using ROC curves and calibration curves of the training and validation sets. Furthermore, the sensitivity and specificity of the new model in assessing the risk of stroke-associated pneumonia in patients undergoing acute ischemic stroke thrombectomy were compared with those of the AIS-APS, A2DS2 score, ISAN score, and PANTHERIS score using ROC curves. The aim of this analysis was to create a reliable model that effectively predicts the occurrence of stroke-associated pneumonia in patients undergoing acute ischemic stroke thrombectomy.

### Statistical method

This study utilized the “glmnet” software package for performing LASSO regression analysis to identify the factors influencing stroke-associated pneumonia in acute ischemic stroke patients undergoing thrombectomy. Subsequently, the “glm” software package was employed to determine the significant risk factors through multiple logistic regression. A nomogram was constructed using the “rms” package in R software to represent the risk of stroke-associated pneumonia in these patients. The model was validated using 500 resampled bootstrap methods and the ROC curve and calibration curve were drawn using the “fbroc” and “rms” packages in R software. The obtained results were then compared with AIS-APS, A2DS2 score, ISAN score, and PANTHERIS score. Moreover, the practical clinical value of the model was evaluated by drawing a clinical decision curve using the “rmda” package.

## Results

This study included a total of 762 patients, with an incidence rate of 44% in the training set and 50% in the validation set. There was no statistically significant difference between the two groups (*p* > 0.05). The table indicated statistically significant differences (*p* < 0.05) in Thrombolysis, D-dimer, FBG, postoperative NLR, and postoperative SII, between the two patient groups, while no other data showed significant statistical differences (*p* > 0.05). Please refer to Table 1 for further details.

**Table 1:** Baseline characteristics of AIS patients in training and validation cohort.

Variables	Validation cohort *n* = 229	Training cohort *n* = 533	*p*-value
Stroke associated pneumonia			0.826
N	126 (55.0%)	287 (53.8%)	
Y	103 (45.0%)	246 (46.2%)	
Demographic characteristics			
Gender			0.916
Female	88 (38.4%)	201 (37.7%)	
Male	141 (61.6%)	332 (62.3%)	
Age			0.218
<70	105 (45.9%)	272 (51.0%)	
≥70	124 (54.1%)	261 (49.0%)	
Comorbidities and disease history			
History of hypertension			0.778
N	93 (40.6%)	209 (39.2%)	
Y	136 (59.4%)	324 (60.8%)	
History of diabetes			0.832
N	184 (80.3%)	423 (79.4%)	
Y	45 (19.7%)	110 (20.6%)	
History of smoking			0.422
N	163 (71.2%)	396 (74.3%)	
Y	66 (28.8%)	137 (25.7%)	
History of COPD			0.636
N	225 (98.3%)	519 (97.4%)	
Y	4 (1.75%)	14 (2.63%)	
History of cerebral infarction			0.005
N	201 (87.8%)	420 (78.8%)	
Y	28 (12.2%)	113(21.2%)	
History of Atrial Fibrillation			0.797
N	160 (69.9%)	379 (71.1%)	
Y	69 (30.1%)	154 (28.9%)	
History of heart failure			1.000
N	217 (94.8%)	506 (94.9%)	
Y	12 (5.24%)	27 (5.07%)	
History of Myocardial infarction			0.123
N	228 (99.6%)	521 (97.7%)	
Y	1 (0.44%)	12 (2.25%)	
Clinical parameters			
Thrombolysis			0.035
N	157 (68.6%)	406 (76.2%)	
Y	72 (31.4%)	127 (23.8%)	
Blockage of blood vessels			0.742
Post-loop	39 (17.0%)	84 (15.8%)	
Pre-cycle	190 (83.0%)	449 (84.2%)	
Difficulty swallowing			0.625
N	101 (44.1%)	247 (46.3%)	
Y	128 (55.9%)	286 (53.7%)	
Hyperperfusion (bleeding or oozing)			0.161
N	177 (77.3%)	437 (82.0%)	
Y	52 (22.7%)	96 (18.0%)	
GCS			0.978
<8	40 (17.5%)	91 (17.1%)	
≥8	189 (82.5%)	442 (82.9%)	
NIHSS			0.837
<16	144 (62.9%)	341 (64.0%)	
≥16	85 (37.1%)	192 (36.0%)	
Time of onset (hour, median [IQR])	4.00 [3.00;7.00]	4.50 [3.00;8.00]	0.236
Body temperature (°, median [IQR])	36.6 [36.5;36.8]	36.6 [36.5;36.8]	0.299
Preoperative Laboratory parameters			
Neu (10^9/L, median [IQR])	5.90 [4.30;8.10]	6.00 [4.40;8.20]	0.799
HB (g/L, median [IQR])	137 [125;147]	136 [123;149]	0.923
PLT (10^9/L, median [IQR])	206 [172;247]	202 [162;245]	0.409
Mono (10^9/L, median [IQR])	0.40 [0.30;0.60]	0.40 [0.30;0.60]	0.376
Lym (10^9/L, median [IQR])	3.50 [2.50;5.00]	1.40 [1.00;2.00]	0.234
LMR (median [IQR])	3.50 [2.42;4.96]	3.40 [2.33;4.83]	0.611
NLR (median [IQR])	3.93 [2.41;7.44]	4.19 [2.47;7.12]	0.507
SII (median [IQR])	782 [466;1,460]	816 [476;1,462]	0.618
PLR (median [IQR])	134 [91.8;201]	140 [100;197]	0.556
SIS (median [IQR])			0.480
0	23 (10.0%)	66 (12.4%)	
1	89 (38.9%)	217 (40.7%)	
2	117 (51.1%)	250 (46.9%)	
FBG (mmol/L,median [IQR])	7.64 [6.49;9.86]	7.12 [6.17;9.04]	0.008
ALB (g/L, median [IQR])	37.4 [34.1;40.2]	37.6 [34.6;41.3]	0.138
CL (mmol/L,median [IQR])	105 [102;107]	105 [102;107]	0.699
Na (mmol/L,median [IQR])	138 [137;140]	139 [137;140]	0.391
PT (s, median [IQR])	13.2 [12.6;13.8]	13.1 [12.6;13.7]	0.350
INR (median [IQR])	1.03 [0.96;1.08]	1.01 [0.96;1.07]	0.315
APTT (s, median [IQR])	33.9 [31.5;36.6]	33.8 [30.9;37.2]	0.813
Fib (g/L, median [IQR])	3.24 [2.68;3.78]	3.19 [2.72;3.78]	0.987
TT (s,median [IQR])	17.8 [17.0;18.8]	17.7 [16.9;18.9]	0.449
D-dimer (mg/L, median [IQR])	1.42 [0.73;2.38]	1.19 [0.57;2.32]	0.016
Postoperatively laboratory parameters			
WBC (10^9/L, median [IQR])	9.10 [7.30;11.1]	8.70 [7.10;10.5]	0.082
Neu (10^9/L, median [IQR])	7.50 [5.60;9.50]	7.00 [5.40;8.80]	0.082
HB (g/L, median [IQR])	125 [113;136]	124 [113;135]	0.670
PLT (10^9/L, median [IQR])	195 [163;229]	191 [155;230]	0.291
Mono (10^9/L, median [IQR])	0.40 [0.30;0.60]	0.43 [0.30;0.60]	0.508
Lym (10^9/L, median [IQR])	1.10 [0.80;1.50]	1.20 [0.80;1.60]	0.138
LMR (median [IQR])	2.50 [1.67;3.86]	2.50 [1.71;3.80]	0.922
NLR (median [IQR])	6.50 [4.03;11.0]	5.92 [3.90;9.22]	0.049
SII (median [IQR])	1,222 [787;2,112]	1,119 [729;1743]	0.039
PLR (median [IQR])	177 [124;241]	164 [122;229]	0.110
SIS (median [IQR])			0.375
0	11 (4.80%)	38 (7.13%)	
1	81 (35.4%)	198 (37.1%)	
2	137 (59.8%)	297 (55.7%)	

AIS, Stroke associated pneumonia; COPD, chronic obstructive pulmonary disease; GCS, Glasgow Coma Scale; NIHSS, National Institutes of Health Stroke Scale; Neu, neutrophil count; HB, hemoglobin; PLT, platelet; Mono, mononuclear leucocyte; Lym, lymphocyte count; LMR, lymphocyte/monocyte ratio; NLR, neutrophil/lymphocyte ratio; SII, platelet*neutrophil/lymphocyte count; PLR, platelet/lymphocyte ratio; SIS, inflammation score, a comprehensive score of ALB and LMR; FBG, fasting blood glucose; ALB, albumin; CL, chloride; Na, sodium; PT, prothronmbin time; INR, international normalized ratio; APTT, activated partial thromboplastin time; Fib, fibrinogen; TT, thrombin time; WBC, white blood cell.

**Figure 2:**
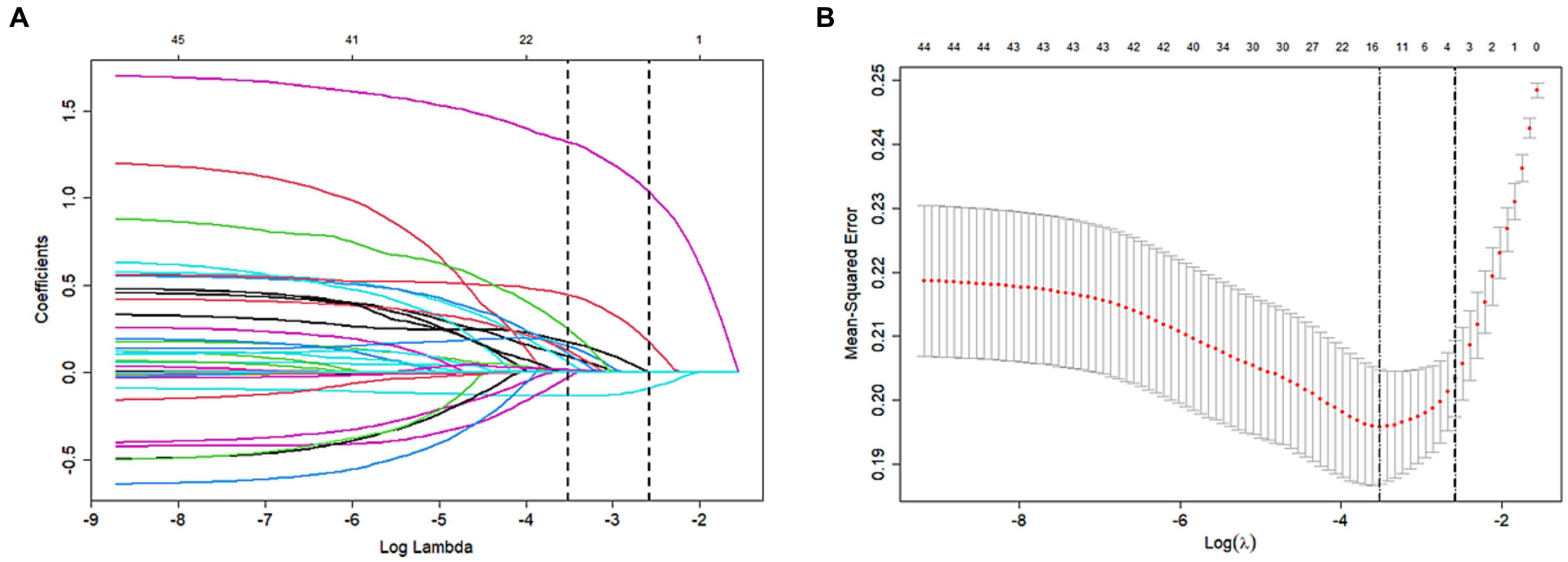
Variable selection by the LASSO binary logistic regression model. **(A)** LASSO coefficient profile of the clinical features. **(B)** The optimal penalization coefficient lambda was generated in the LASSO via tenfold cross-validation. We plotted the partial likelihood deviance (binomial deviance) curve versus log (lambda) and drew dotted vertical lines based on 1 standard error criteria. LASSO Least absolute shrinkage and selection operator.

**Figure 3:**
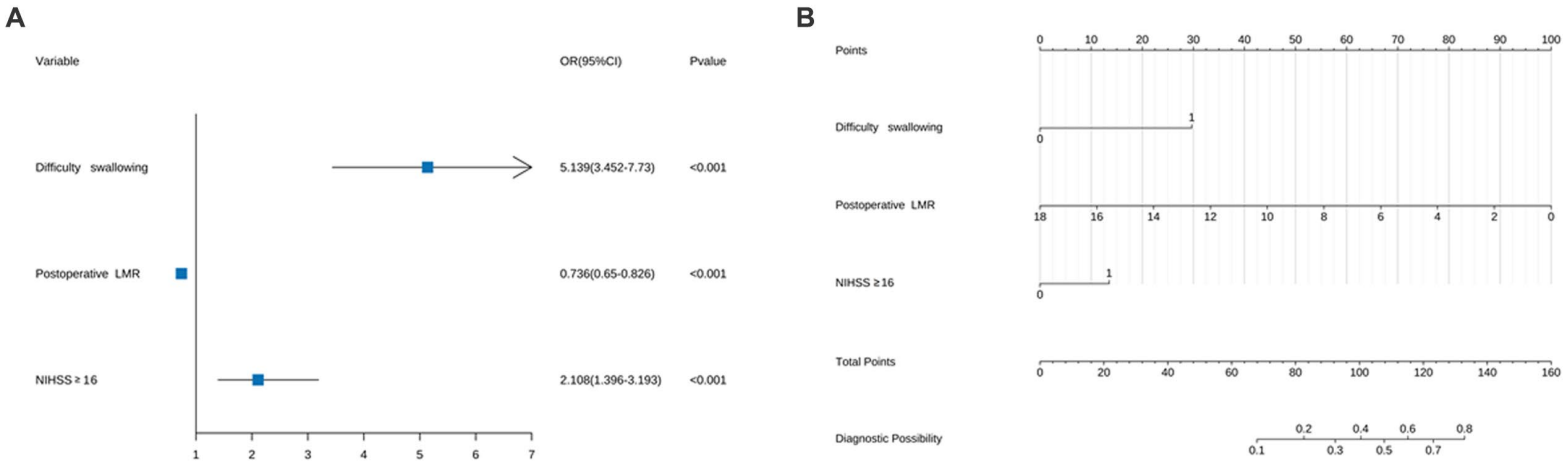
**(A)** Forest map of pneumonia risk in patients with acute ischemic stroke thrombectomy and **(B)** column chart of pneumonia risk in patients with acute ischemic stroke thrombectomy. For all patients, add up the scores of the three indicators, and the total score is located on the “Total Points” axis, which corresponds to the risk of SAP in patients with acute ischemic stroke thrombectomy.

The influencing factors were identified through LASSO regression analysis, as depicted in Figure 2. The results of the multiple logistic regression analysis demonstrated that an NIHSS score of ≥16 points (OR 2.11; 95% CI 1.40–3.19; *p* < 0.001), postoperative LMR (OR 0.74; 95% CI 0.65–0.83; *p* < 0.001), and dysphagia (OR 5.14; 95% CI 3.452–7.73; *p* < 0.001) were found to be statistically significant risk factors for SAP, as demonstrated in Figure 3.

A predictive model for the risk of associated pneumonia in patients undergoing thrombectomy for acute ischemic stroke was established by creating a column chart based on NIHSS scores ≥16, postoperative LMR, and dysphagia. The chart shows the scores and risks of different risk factors in various line segments, where each risk factor corresponds to different risk scores. The total score reflects the overall risk of pneumonia occurrence, with higher scores indicating a higher likelihood of pneumonia, as illustrated in Figure 3.

**Figure 4:**
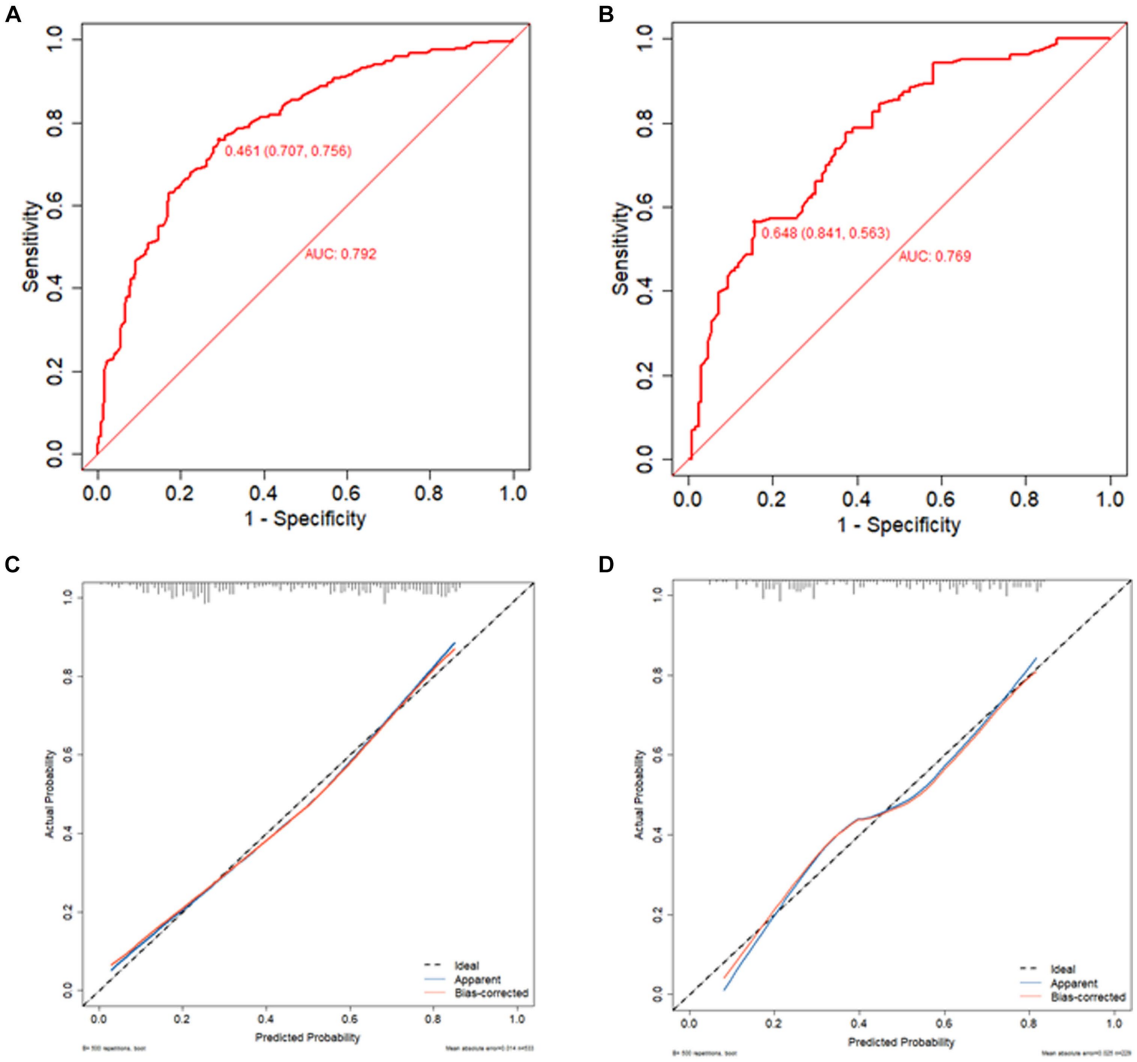
ROC curve of training set **(A)**, ROC curve of validation set **(B)**, calibration curve of training set **(C)**, and calibration curve of validation set **(D)**. The red line indicates the correlation between actual and predicted values. The diagonal dashed line represents the most perfect prediction, and the cross feature represents the correction between the predicted value and the actual value. *p* > 0.05, good fit.

The predictive model developed in this study aimed to assess the risk of associated pneumonia in patients who underwent thrombectomy for acute ischemic stroke. The area under the receiver operating characteristic (ROC) curve was 0.792 (95% CI 0.754–0.83) for the training set and 0.769 (95% CI 0.708–0.829) for the validation set, indicating good discriminative ability. In the calibration chart, the red line represents the correlation between actual and predicted values. The diagonal dashed line represents a perfect prediction, and the cross feature indicates the deviation between the predicted and actual values. The Hosmer-Lemeshow goodness-of-fit test revealed X-squared values of 6.0678 (*p* = 0.6396) for the training set and 12.008 (*p* = 0.1509) for the validation set, suggesting a good fit as the *p* > 0.05 indicated. The calibration curves for both the training and validation sets closely resemble the standard curve, indicating strong discrimination, calibration, and predictive value, as shown in Figure 4.

**Figure 5:**
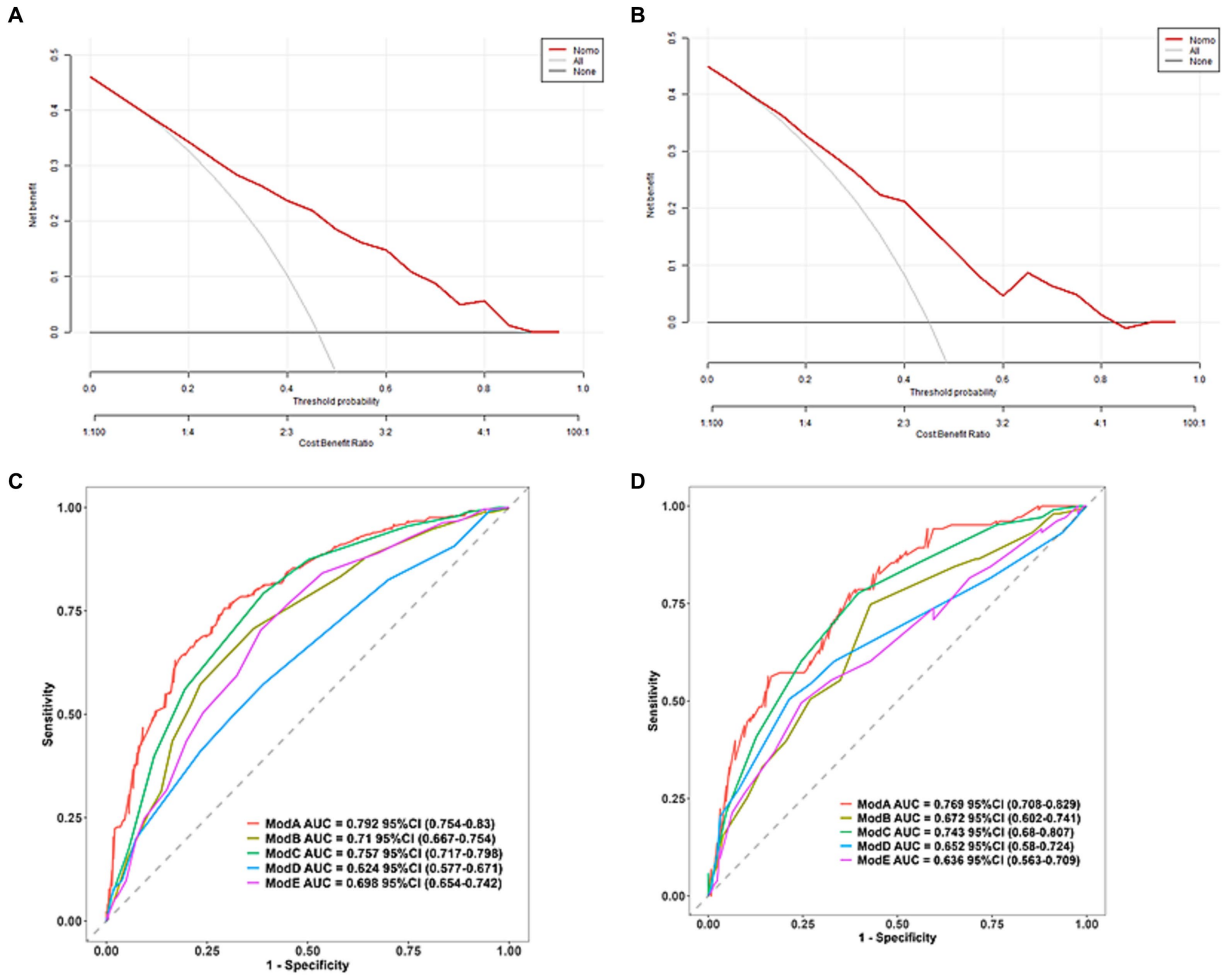
Clinical decision diagrams for training set **(A)** and validation set **(B)**. The net benefit is calculated by adding true positives and subtracting false positives. For cases where the threshold probability is greater than 7%, applying a column chart will increase net benefits compared to treating all strategies or not treating strategies. **(C)** Predicts the training set ROC curve of SAP using different scoring systems and **(D)** predicts the validation set ROC curve of SAP using different scoring systems (Model A: column chart, Model B: ISAN score, Model C: A2DS2, Model D: PANTHERIS, Model E: AIS-APS).

The clinical decision curve and column chart demonstrate high net benefits in both the training and validation sets. Within the threshold probabilities ranging from 7 to 90%, the column chart outperforms the “treat-all” or “no-treatment” strategies, resulting in increased net benefits, as illustrated in Figure 5.

Further predictions of the incidence of associated pneumonia in patients with acute ischemic stroke thrombectomy were performed using other scoring methods. The AIS-APS scoring method had AUCs of 0.698 (95% CI 0.654–0.742) and 0.636 (95% CI 0.563–0.709) for the training and validation sets, respectively. For the A2DS2 scoring method, the AUCs were 0.757 (95% CI 717–0.798) for the training set and 0.743 (95% CI 0.68–0.807) for the validation set. The ISAN scoring method had AUCs of 0.684 (95% CI 0.62–0.749) and 0.672 (95% CI 0.602–0.741) for the training and validation sets, respectively. The AUCs for the PANTHERIS scoring method were 0.624 (95% CI 0.577–0.671) for the training set and 0.652 (95% CI 0.58–0.724) for the validation set, as shown in Figure 5. The discrimination of this model is higher compared to the AIS-APS, A2DS2 score, ISAN score, and PANTHERIS score.

## Discussion

Stroke-associated pneumonia is a common complication in clinical practice following a stroke (19). Previous research reports have primarily focused on patients with cerebral hemorrhage or cerebral ischemia, without specifically screening out patients who underwent thrombus removal (16, 21). In a study by Yan et al., it was discovered that the probability of stroke-associated pneumonia occurring in patients with cerebral hemorrhage is 25.52% (10). Furthermore, the probability of pneumonia occurring in high-risk populations can be as high as 40% (21). In a cohort study conducted in England and Wales, patients with ischemic or hemorrhagic stroke admitted to the stroke unit were included. It was found that the median prevalence of stroke-associated pneumonia (SAP) was 8.5%, with the highest recorded prevalence being 21.4% (22). The data from this study suggests that the incidence of stroke-related pneumonia in patients after thrombectomy is 45.8%, which is higher than the incidence reported in other research studies. This discrepancy may be attributed to the fact that our sample consisted of patients who underwent thrombectomy for acute ischemic stroke. Following thrombectomy, there is a risk of brain tissue ischemia–reperfusion injury, as well as stress-induced immune responses triggered by surgery and contrast agents. In a separate study, a rare complication of contrast agent encephalopathy was observed after carotid artery stent surgery (23). The underlying pathogenesis of this complication is currently unclear, but it may be associated with chemical toxicity and brain edema caused by the passage of contrast agents through the blood–brain barrier (23). All of these factors have the potential to alter the patient’s immune and inflammatory response, thus promoting the occurrence of SAP.

Acute ischemic stroke-associated pneumonia was initially proposed by Professor Hilker et al. (24). When patients experience acute ischemic stroke, it can result in immune suppression, and the incidence of aspiration may also contribute to the development of SAP. When brain tissue ischemia occurs, it can trigger a series of immune inflammatory reactions, leading to the production of toxic substances, such as inflammatory cytokines, chemokines, and reactive oxygen species. These substances can disrupt the blood–brain barrier. They can rapidly activate immune inflammatory cells, affecting their entry into the ischemic area and triggering immune responses that further damage neurons (25). In clinical practice, the primary diagnostic indicators for SAP include WBC, Neu, PCT, CRP, and other markers. Various studies have demonstrated that peripheral blood inflammatory markers, such as LMR, NLR, and SII, are closely associated with stroke-associated pneumonia, providing a scientific and theoretical foundation for the diagnosis and prediction of this condition (26). For example, Nam et al. discovered that NLR is correlated with the severity of SAP (27). LMR reflects the immune response of the body, and previous studies have mostly demonstrated it to be one of the prognostic factors for many cancers (28). In this study, it was found that LMR is one of the influencing factors in the development of SAP in patients. Therefore, in clinical practice, the dynamic monitoring of LMR can be used for early prevention, ultimately reducing the incidence of stroke-associated pneumonia.

Meanwhile, the degree of impairment in consciousness of stroke patients is closely related to the occurrence of stroke-associated pneumonia (29). In the acute phase of ischemic stroke, patients are susceptible to neurological and other complications. The NIHSS scoring system is a commonly utilized scale for assessing the severity of neurological damage in patients (30). The score covers various aspects, including the patient’s level of consciousness, gaze, visual acuity, facial paralysis, upper and lower limb movement, limb ataxia, sensation, language, articulation disorders, and neglect. A higher NIHSS score indicates more severe nerve damage. When consciousness disorders worsen, it can inhibit the cough reflex, leading to weakened respiratory movement or even ventilator paralysis. This can result in poor drainage of respiratory secretions and lead to pneumonia. Additionally, studies have shown that while 45% of normal individuals can experience trace inhalation of oral secretions while falling asleep, the probability of this occurring increases to 90% in patients with consciousness disorders. In their research, Chumbler et al. found that the NIHSS score has high predictive significance for the occurrence of stroke-associated pneumonia (31). In this study, swallowing difficulties were identified as a risk factor. Stroke-induced damage to the swallowing center of patients disrupts the control ability of the pharyngeal muscles and nerves, resulting in swallowing dysfunction. This dysfunction increases the likelihood of foreign objects entering the airways and bronchi, thereby increasing the probability of pneumonia occurrence (32). Evaluating the patient’s swallowing function, providing effective oral care, and conducting early rehabilitation exercises are measures that can reduce the risk of associated pneumonia, as shown by prior research (33).

Nonetheless, there are still limitations to this study as the collected data is limited to our hospital and has not yet been clinically validated by external hospitals. Additionally, our prediction model is built based on the clinical indicators we can collect, and it is not ruled out that there are better clinical indicators to help us predict SAP. The heterogeneity of the study population should be acknowledged so that future work is needed to explore how subgroups of patients can have different results/conclusions (34). What’s more, preventing SAP and advancing the treatment window for stroke-associated pneumonia to the ultra-early stages of stroke occurrence are important issues that require further exploration and study.

In conclusion, the nomogram chart that we constructed based on NIHSS≥16, postoperative LMR, and dysphagia as three influencing factors, has shown good discrimination and calibration in predicting the risk of associated pneumonia in patients with acute ischemic stroke thrombectomy. It is noteworthy that the predictive accuracy of this chart surpasses that of the AIS-APS, A2DS2 score, ISAN score, and PANTHERIS score.

## Data availability statement

The raw data supporting the conclusions of this article will be made available by the authors, without undue reservation.

## Ethics statement

The studies involving human participants were reviewed and approved by the Ethics Committee of Taizhou Enze Medical Center (Group) (Ethics Number: K20201104). Written informed consent was not required as per local legislation and institutional requirements.

## Author contributions

JYW: Writing – original draft, Data curation. CY: Data curation, Writing – original draft. RHZ: Data curation, Writing – original draft. WH: Writing – review & editing, Validation. PY: Formal analysis, Supervision, Writing – original draft. YQJ: Investigation, Visualization, Writing – original draft. WJH: Methodology, Writing – review & editing. RFS: Writing – original draft, Project administration, Supervision. YPJ: Funding acquisition, Writing – review & editing, Conceptualization, Writing – original draft.
